# Genome characterization of *Salmonella enterica* serovar Enteritidis phage SESL from a Malaysian hot spring salt lick

**DOI:** 10.1128/mra.00061-26

**Published:** 2026-04-20

**Authors:** Yee Fan Tan, Prasanna Mutusamy, Thomas Sicheritz-Ponten, Bent Petersen, Kumara Thevan, Heera Rajandas, Sivachandran Parimannan

**Affiliations:** 1 Center of Excellence for Omics-Driven Computational Biodiscovery (COMBio), AIMST University 649161, Bedong, Kedah, Malaysia; 2 Center for Evolutionary Hologenomics, Globe Institute, University of Copenhagen 552766, Copenhagen, Denmark; 3 Faculty of Agro Based Industry, University of Malaysia Kelantan 356748 , Jeli, Kelantan, Malaysia; Queens College, Queens, New York, USA

**Keywords:** phage genome, Malaysia, hot spring, *Salmonella* Enteritidis

## Abstract

The complete genome of *Salmonella enterica* serovar Enteritidis phage SESL, isolated from a Malaysian hot spring salt lick sample, belonging to genus *Tequintavirus*, is 111,296 bp in length and has a GC content of 39.26%. SESL has 161 protein-coding genes and shares 99.99% nucleotide similarity with *Salmonella* phage STP-SP2.

## ANNOUNCEMENT

*Salmonella* phages are promising alternatives to antibiotics for controlling multidrug-resistant strains in poultry and food systems (1, 2). Here, we announce the genome of phage SESL, isolated from a water sample collected at a hot spring salt lick in Kelantan, Malaysia (5.6635°N, 101.7166°E).

Phage isolation was performed using *S*. *Enteritidis* strain CS/SEN18 obtained from the Center of Excellence for Omics-Driven Computational Biodiscovery (COMBio) in-house culture collection as the host. Enrichment was performed by mixing 10 mL of Luria-Bertani (LB) broth (Oxoid, UK), 200 µL of the filtered water sample, and 100 µL of overnight host culture. The mixture was incubated at 37°C for 24 h and centrifuged for 10 min at 4°C. The lysate was filtered (0.22 µm) and subjected to triple plaque purification (3) following 10-fold serial dilutions (10^−1^–10^−6^) in LB. DNA extraction from high-titer phage stock (10^8^ PFU/mL) was done using phenol-chloroform method (4) and quantified using Qubit 4 fluorometer (Cat. No. Q33226, Thermo Fisher). DNA libraries were prepared using the NEBNext Ultra II DNA Library Preparation Kit (Cat. No. E7645, New England Biolabs), and sequencing was done on an Illumina NovaSeq 6000 platform with paired-end 2 × 150 bp reads. A total of 5,609,210 paired-end reads were generated. Raw read quality was assessed using FastQC v0.12.1 (5) before being trimmed by BBDuk (BBMap v38.18 package) with parameters: ktrim=r hdist=1 tpe tbo minlen=100 qtrim=rl trimq=28 (https://github.com/BioInfoTools/BBMap/blob/master/docs/guides/BBDukGuide.txt). Subsequent bioinformatic analysis used default parameters unless otherwise specified. 50,000 reads were subsampled using seqtk (https://github.com/lh3/seqtk) and assembled into a single contig through Unicycler v0.5.0 (6). CheckV v0.7.0 using an AAI-based method determined that the assembled genome is of high quality and has a completeness of 98.48%, with a high confidence level (1.25% error) (7). PhageTerm 4.1.0 detected 10,186-bp direct terminal repeats (DTRs) (8).

**Fig 1:**
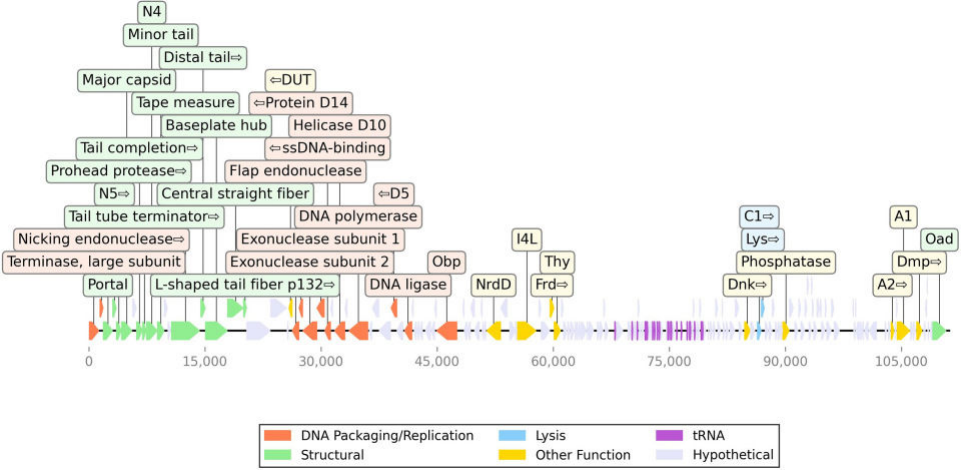
Linear genome map of *Salmonella* phage SESL. The genome is 111,296 bp in length. Arrows represent predicted coding sequences (CDS) and are color-coded by function: structural (green), DNA packaging/replication (coral), lysis (blue), tRNA (purple), other functional (yellow), and hypothetical (gray). The map was generated using DNA Features Viewer.

Phage SESL is a complete, linear, double-stranded DNA sequence with a final length of 111,296 bp, an average fold coverage of 127.2, and a GC content of 39.26%. The annotation of the genome was carried out with Prokka v1.14.6 (9) in conjunction with the PHROGs database (10). A total of 22 tRNAs and 161 coding sequences (CDSs) were successfully annotated, of which 38 were known functional or putative genes, and 123 were hypothetical proteins. The genomic organization, including functional modules for structure, packaging, and lysis, is visualized in Fig. 1. The genome was analyzed using PhageLeads (11), which did not show the presence of predicted temperate lifestyle genes, antimicrobial resistance genes, or virulence genes in SESL. Basic Local Alignment Search Tool (BLASTn; https://blast.ncbi.nlm.nih.gov/Blast.cgi) search of the complete genome against the core_nt NCBI database (accessed 14 January 2026) revealed that *Salmonella* phage STP-SP2 (GenBank accession number OR485018.1) was the most similar match, sharing 99.99% nucleotide identity. Based on this similarity and the presence of large DTRs, SESL is classified within the genus *Tequintavirus*.

## Data Availability

The complete genome sequence of phage SESL has been deposited in GenBank under the accession number PQ041310.1. The associated BioProject, SRA, and BioSample accession numbers are PRJNA1136242, SRX25337423, and SAMN42510465, respectively.
